# Nematode Genome Announcement: A Draft Genome of Seed Gall Nematode, *Anguina tritici*

**DOI:** 10.2478/jofnem-2023-0031

**Published:** 2023-11-15

**Authors:** Ashish Kumar Singh, Antara Das, Ila Joshi, Manish Kumar, Deshika Kohli, Pankaj, Kishor Gaikwad, Pradeep Kumar Jain, Anil Sirohi

**Affiliations:** Division of Nematology, ICAR-Indian Agricultural Research Institute, Pusa Campus, New Delhi-110012, India.; Division of Crop Protection, ICAR-Vivekananda Parvatiya Krishi Anusandhan Sansthan, Almora, Uttarakhand 263601, India.; ICAR-National Institute for Plant Biotechnology, Pusa Campus, New Delhi, 110012, India.

**Keywords:** *Anguina tritici*, earcockle, tundu disease, genome sequence, Illumina MiSeq

## Abstract

*Anguina tritici* is the first plant-parasitic nematode described in literature, dating back to the year 1743. It is responsible for causing earcockle (seed gall) and tundu diseases in wheat and rye. Notably, this nematode has been observed to survive in an anhydrobiotic state for up to 32 years within wheat seed galls. These exceptional characteristics have inspired the sequencing of the *A. tritici* genome. In this study, we present the initial draft genome of *A. tritici*, obtained using the Illumina MiSeq platform with coverage of 60-fold. The genome is estimated to have a size of 164 Mb and comprises 39,965 protein-coding genes, exhibiting a GC content of 39.1%. The availability of this genome data will serve as a foundation for future functional biological investigations, particularly for genes whose functions remain unknown to this day.

At least four distinct major lineages of plant parasites have emerged within the species-rich and trophically diverse phylum Nematoda (Quist et al., 2015). *A. tritici*, commonly known as the seed gall nematode, is a plant parasitic nematode (PPN) that infects the seeds of wheat and barley, leading to the development of the earcockle disease. This nematode also acts as a vector for the bacteria *Clavibacter tritici*, causing tundu disease in their association (Swarup and Gupta, 1971). According to the phylogenetic analysis conducted by van Megen et al. (2009), *Anguina tritici* is classified within the Anguinidae family, along with its closely related species *Ditylenchus dipsaci* and *D. destructor*. In the past decade, the genomes of several parasitic nematodes have been sequenced, enabling comparative genomic analyses that shed light on the gain or loss of novel genes among nematode species (Wasmuth et al., 2008). As more genome sequences become available, researchers will be able to identify species-specific peculiarities in biological characteristics.

The biology of *A. tritici* differs from other PPNs, which usually feed on or in the roots of the crop plants but do not infest the seeds. *A. tritici* is an obligate parasite specifically targeting the seeds of its host plant wheat and barley, unlike the rice white tip nematode, *Aphelenchoides besseyi*, which also survives in an anhydrobiotic state but in dry tissues, under hulls of rice grains. *A. tritici* exhibits the remarkable ability to survive for several decades in an anhydrobiotic state without losing its parasitic capability (Limber, 1973) but *A. besseyi* is reported to survive for a maximum of three years without losing its parasitic ability (Cralley, 1949; Yoshi and Yamamoto, 1950) Among plant parasitic nematodes, *A. tritici* exhibits notable variability in certain biological traits, distinguishing it from the other PPN species and making it an attractive model system for investigating fundamental questions related to survival, ageing, seed parasitism, bacterial associations, and more. Currently, limited genomic information is available regarding plant-parasitic nematodes that specifically target crop seed.

Recently, the genome of *Aphelenchoides besseyi*, a nematode that feeds on the growing tips of rice seedlings, causing the white-tip disease, has been published (Lai et al., 2023; Ji et al., 2023). However, unlike *A. tritici*, it does not enter the grain but remains localized in the husk part of the paddy seed. The genome sequences of two closely related species of *Anguina tritici*, namely *Ditylenchus dipsaci* (Mimee et al., 2019) and *D. destructor* (Zheng et al., 2016) are also available. These genome sequences provide valuable resources for comparative analysis and serve as important references for understanding the genomic features of *A. tritici* in a broader context. The objective of this study was to present the draft genome of the seed gall nematode, *A. tritici*. To propagate the *A. tritici*, cockled seeds were crushed and placed in a modified Baermann funnel for the extraction of second-stage juveniles (J2s). The extracted J2s were then inoculated onto greenhouse-grown wheat cultivar ‘PBW 343’ for multiple generations to generate an inbred population of the nematode. Before DNA extraction, J2s were surface sterilized using M9 buffer and nematode sterilization buffer to remove any contaminants (Joshi et al., 2019). High-quality genomic DNA was extracted using the modified fast CTAB method (Thomas et al., 1997). The isolated high-quality DNA was utilized for the preparation of a genomic DNA sequencing library using the TruSeq DNA sample preparation kit from Illumina technologies, USA by following the manufacturer’s protocol. The quality of DNA libraries was assessed using the Bioanalyzer 2100 from Agilent technologies, USA. Paired-end sequencing of the genomic library was performed on the Illumina MiSeq (2X300) platform. The genome size was estimated using the K-mer value of the Abyss Version 1.3.7 (Simpson et al., 2009). Paired-end files were merged using PandaSeq 2.5 (Masella et al., 2012), and the reads were subjected to quality checking using FastQC software v3.0 (Andrew, 2012). High-quality reads with a Phred score of ≥20, after trimming adapters using trimmomatic version 0.39 (Bolger et al., 2014), were retained. *De novo* assembly was carried out using Abyss Version 1.3.7 (Simpson et al., 2009) at different K-mers (45, 50, 55, and 61). The quality assessment of the assembled genome was performed using BUSCO version 3.0.2 (Robert et al., 2017). Protein-coding genes were predicted using the *ab initio* approach of AUGUSTUS version 3.0.3 (Stanke et al., 2004) with default parameters. Predicted genes with a length of less than 400 bp were excluded, and the remaining genes were annotated against the NCBI redundant nucleotide database using RAPsearch2 and an in-house perl script (Yuzhen et al., 2011).

The Illumina MiSeq sequencing strategy generated a total of 11,065,381,294 bases, with a read count of 40,210,848. The GC percentage, Q20 percentage, and Q30 percentage were calculated as 39.1%, 92.73%, and 84.08%, respectively. By merging the paired-end reads using the Pandaseq program, a total of 7,938,027,993 bases and 18,913,374 read counts were obtained, with a GC percentage of 39.1%. The draft genome assembly of *A. tritici* resulted in a size of up to 164 Mb with a GC content of 39.1%. The genome sequence data has been submitted to GenBank with the following identifiers: BioProject ID: PRJNA802691, BioSample: SAMN25565353, and accession ID: JAKMXJ000000000. The genome achieved a total coverage level of 60-fold. Quantitative assessment of assembly completeness was performed using BUSCO, revealing C: 36.6% [S: 35.5%, D: 1.1%], F: 14.8%, M: 48.6%, and n: 982 (Table 1). A total of 44,954 protein coding genes were predicted using the *ab initio* Augustus version 3.0.3. After filtering genes with a length below 400 base pairs, a total of 39,965 predicted genes were identified, resulting in an overall gene density of 26.5784. The average gene length was 1,347 bp, while the average exon and intron lengths were 168 bp and 280 bp, respectively. Of the 41,746 predicted transcripts, 62% were successfully annotated with functional annotations against the NCBI database using RAP search and an in-house perl script. However, 14,856 (37.18%) genes remained unannotated in the NCBI database. In comparison with closely related species, the genome size of *Anguina tritici* (164 Mb) falls between that of *Ditylenchus dipsaci* (227.2 Mb) (Mimee et al., 2019) and *Ditylenchus destructor* (112 Mb) (Zheng et al., 2016) indicating that *Anguina tritici* has a moderately sized genome in comparison to the other two species. The GC content, shows slight variations among the three species. *Anguina tritici* has the highest GC content, indicating a relatively higher abundance of G and C nucleotides in its genome compared to the *Ditylenchus dipsaci* (37.5) and *Ditylenchus destructor* (36.6). *Anguina tritici* has the highest number of coding genes (39,965) among the three species, indicating a more complex and diverse genetic repertoire. *Ditylenchus dipsaci* has an intermediate number of genes (26,428), while *Ditylenchus destructor* has the fewest genes (13,938). These variations may reflect evolutionary adaptations and functional differences between these nematode species.

**Table 1.** Characteristics of *A. tritici* genome.

**Number of contigs**	**843604**
**Assembled genome size**	164398910 (164 Mb)
**N50**	3232
**Number of gene models**	39965
**Median intron, bp**	65
**G+C %**	39.1%
**Gene density**	26.5784
**Average gene size (bp)**	1347
**Average CDS length (bp)**	168.88
**Average number of exon/genes**	3.18
**Average intron length (bp)**	280
**BUSCO**	C: 36.6% [S: 35.5%, D: 1.1%], F: 14.8%, M: 48.6%, n: 982

Broadly, the genome size of *A. tritici* was determined to be 164 Mb, which is larger than most of the published genomes of PPNs, except for *Meloidogyne arenaria*, *Rotylenchulus reniformis*, and which are 284 Mb and 380 Mb, respectively (Nyaku et al., 2014; Sato et al., 2018). The GC content was measured to be 39.1%, which is higher than most PPNs, except for *Bursaphelenchus xylophilus* (Kikuchi et al., 2011). From the *A. tritici* genome, a total of 39,965 protein-coding genes were predicted. This number is higher than most of the PPN genomes sequenced, except for *Meloidogyne javanica*, *Meloidogyne floridensis*, and *Meloidogyne arenaria* (Blanc-Mathieu et al., 2017). On an average, three exons per gene were observed, compared to two exons per gene in *Pratylenchus coffeae* (Burke et al., 2015) and four in most PPNs. The *A. tritici* genome exhibits characteristics of a compact parasitic genome, similar to other nematodes, with a low number of small introns and a high number of repeats. Out of the 41,746 predicted transcripts, 62% were successfully annotated with functional annotations. These unannotated genes may possess unique functions in the *A. tritici* genome since they do not show significant matches in the NCBI database. Their validation could shed light on their species-specific roles. The *A. tritici* genome will be valuable in unravelling the mechanisms involved in anhydrobiosis and the genes responsible for parasitizing the seed parts of plants, an area of nematology that has been less explored. Integrating RNAseq data in the annotation process could enhance gene prediction and annotation accuracy, thereby improving the overall quality and completeness of the final genome assembly. Future studies should consider incorporating RNAseq data to further enhance the understanding of the *A. tritici* genome.
